# The impetus to Africa CDC’s mandate in curbing the rising trend of Antimicrobial Resistance (AMR) in Africa: the launch of the Africa CDC AMR surveillance network during the 8th advanced course in diagnostics (ACDx)

**DOI:** 10.11604/pamj.2017.28.271.14388

**Published:** 2017-11-28

**Authors:** Noah Fongwen Takah, Priyanka Shrestha, Rosanna Peeling

**Affiliations:** 1 The International Diagnostics Centre, Clinical Research Department, London School of Hygiene and Tropical Medicine, Keppel Street, London, United Kingdom; 2 Ministry of Public Health, Cameroon

**Keywords:** Antimicrobial resistance, surveillance, network, diagnostics

## Abstract

The rise of antimicrobial resistance is a global threat and Africa like any other developing setting is heavily affected. As one of its missions, the Africa CDC is poised to ensure this rising trend takes a diminishing route in the few years ahead. Diagnostics obviously play a pivotal role in AMR control and the advanced course in diagnostics (ACDx) has been instrumental in training critical decision makers over the past 7 years. This commentary presents an analysis of discussions and exchanges during the launch of the Africa CDC AMR surveillance network at the ACDX and the way forward for its implementation. The diagnostics priorities are also highlighted.

## Commentary

The emergence of “Superbugs” that are resistant to almost all conventional antibiotics clearly presents as one of the most daunting global health challenges in the 21^st^ century [1]. Given the crucial role of antibiotics in preventing the spread of infectious diseases and reducing the rate of mortality among patients in clinics, care homes and intensive care units, the problem of antimicrobial resistance (AMR) has attracted global attention from governments and international health agencies in both developed and developing settings [2]. Inferring from the Lord Jim O'Neil report on AMR, as many as 700,000 people die from AMR annually Worldwide with a projected global annual mortality rate of 10 million by 2050 if nothing urgent is done [3]. There are fears of an apocalyptic picture in a do-nothing scenario in which we might revert to the pre-antibiotic era [3].

The international response has been unprecedented and encouraging. While there are ongoing efforts towards the establishment of a global fund for AMR, the World Health Assembly (WHA) in May 2015 endorsed a global action plan to tackle AMR and has called on governments and private sector commitments towards mitigating the AMR crisis [4]. Consequently, the World Health Organization (WHO) in February 2017 has published a list of bacteria of which urgent antibiotics are needed in a bid to guide Research and Development (R&D) [5] and have developed the GLASS(Global Antimicrobial resistance Surveillance System) to which countries are expected to report the state of AMR [6]. Furthermore, in Africa, the Africa CDC is committed to curb the rising trend of AMR in the continent through the establishment of a surveillance network in the continent that can effective respond to the AMR threat [7]. The International Diagnostics Centre(IDC) of the London School of Hygiene and Tropical Medicine(LSHTM) and the Fondation Merieux have been pivotal in the training of policy makers through the Advanced Course in Diagnostics(ACDx), on critical decision in diagnostics for the past 7 years.

This year's 8^th^ session was unique because it was dedicated to AMR. Taking into account the pervasiveness of diagnostics in tackling AMR and that the ACDx is a forum where experts and policy makers from academia, industry, government and other private sector agencies converge, there was no better opportunity to launch the Africa CDC AMR Surveillance Network (AMRSNET). This diverse gathering was an essential starting point in the creation of sustainable public private partnerships that can drive and support the AMRSNET in fulfilling its goals and challenges. The objectives of the launch of the Africa CDC Antimicrobial resistance (AMR) surveillance network was to enable participants to learn about the Africa CDC AMR Surveillance Network and how the network should function based on lessons learnt from other global, regional and country AMR strategies and policies as well as to brainstorm on the way forward for the use of diagnostics to combat AMR. This commentary presents an analysis of some of the exchanges that took place during the launch of the Africa CDC AMRSNET and the way forward for its implementation, taking into the consideration some overarching diagnostic priorities.

In a roundtable discussion with AMR experts from five African countries, immediate actions were obtained which will be very instrumental in defining specific in-country targets in the national strategic plans. From this roundtable discussion, it was clear that not all countries are at the same level in the control of AMR. Africa CDC would need to take this into consideration when implementing its plan on AMR in the African continent. The findings from the roundtable and the perceived challenges are shown in Table 1. Lessons learnt will also be crucial in guiding in-country implementation of AMR control. Two important lessons would need to be learnt from ongoing experiences in public health implementation in Africa. Firstly, there would be need for an economic case to be made to ensure governments can adopt the AMR strategy. With funding from the UNITAID, the London School of Hygiene and Tropical Medicine carried out an economic evaluation to show the cost effectiveness of introducing and adopting quality assured diagnostics. The final costs on the health system and the government if there is no quality assured testing was shown to be huge [8].

**Table 1:** Immediate actions and perceived challenges from the African country representative

Country	Actions on AMR control so far	Immediate actions	Perceived challenges
Cameroon	No national action plan. Low scale AMR awareness campaigns carried out. Survey of use of antimicrobials carried with the Global Health Systems Solutions (GHSS) in collaboration with the Amsterdam Institute for Global Health.	The setting up and stepwise implementation of the WHO-recommended laboratory-based surveillance system adopted to local AMR detection and local disease burden. The concomitant formulation and implementation of a multisectoral AMR control policy embracing the One Health concept. The development of financial plans and business models to sustain AMR diagnostics and control, and to reduce out of pocket expenses	Funding to step up and implement the laboratory based surveillance system. Slow government “buy-in” for the development and approval of guidelines and policies for AMR in the country. Bringing the related ministries and setting up a legal framework for enforcement of the AMR policies.
Kenya	National action plan developed. National policy document signed by relevant ministries	Develop and disseminate appropriate messages on AMR to suit the different target groups. Enhance multisectoral communication and provide budgetary support. To build the capacity of the laboratories to support AMR surveillance. To review and develop guidelines for Infection Prevention and Control, Hygiene and Sanitation.	Financial and technical support to implement the national action plan. Sustained multisectoral collaboration is a challenge.
Tanzania		Build capacity on AMR surveillance. Implement a stepwise approach that will involve training of AMR mentors/fellows that can enforce the implementation of AMR stewardship in clinical, veterinary and agricultural sectors. Support implementation of national AMR surveillance plan Conduct AMR survey at selected health facilities. The technology and facilities of ongoing global AMR surveillance surveys such as the Global PPS can be leveraged for Tanzania.	Inadequate funding and skilled human resource to conduct large scale national AMR surveys. There is no system in place to ensure real time sharing of AMR data.
Zimbabwe	National plan for AMR launched. The implementation of plan not yet ongoing.	Implement AMR stewardship programmes in health care facilities. Carry out education and awareness of AMR reduction activities. More knowledge attitude and practices surveys need to be conducted in order to guide the content the content of the AMR messages delivered to the public. Build and economic case to convince the governments on the cost-effectiveness of AMR interventions	Clinicians, nurses and laboratory staff don’t interact well enough with regards to antimicrobial prescription to patients. Many clinicians and allied health professionals are not trained on how they can collaborate to promote AMR stewardship. Human resource is challenge. More staff trained in AMR stewardship will be needed to facilitate implementation. Microbiology laboratories have been neglected in the country due to huge focus on HIV and TB. The current HIV and TB vertical programmes may not want to collaborate with other programmes on enabling a wider control. Human resource is challenge. More staff trained in AMR stewardship will be needed to facilitate implementation.
Senegal	National action plan under development	Change behaviour of pharmacists. Prevent over the counter sales of antibiotics. Control the use of antibiotics in animals. Increase awareness of the population through education and sensitization on the consequences of antibiotics misuse.	

This economic case was instrumental in convincing the government of Zimbabwe in engaging in the implementation and scale up of quality assured diagnostics. A similar economic case would be needed alongside a business model to ensure the adoption of the surveillance network by the government. Such a business model will be crucial for the sustainability of the AMR surveillance network in the country. Secondly, there is need to conduct a survey on the current state of antibiotics consumption in the continent. The Global Point Prevalence survey would be a good starting point. Global Point Prevalence Survey is the initiative of Professor Herman Goossens of the Institute of Tropical Medicine, Antwerp, Belgium. This survey, with funding from the European Council, is an innovative strategy to assess global antimicrobial consumption and resistance in hospital settings. It utilizes a simple and sustainable Web-based point system for instant report of hospital data on antimicrobial consumption [9]. Data are collected using two forms: a ward form and a patient form which provide information of the kind of antibiotics used, dose, reason for prescription and if they were in compliance with the guidelines, the use of antibiotics for empirical or targeted therapy and use of biomarkers such as C-reactive protein or pro-Calcitonin. These data are then fed into the web based system for local analysis of findings to inform appropriate clinical practice. This system has the following benefits for AMR surveillance: increase awareness among health personelle on use of antibiotics, building a team where all health personelle work together in achieving a common goal, and providing a quantifiable quality target for improved antimicrobial prescribing [9]. One key limitation of the system is that it considers only clinical antimicrobial resistance control and does not include veterinary and agriculture sectors. In addition, there are challenges on how to standardize human and animal data.

However, the developers have confirmed that the system can be adapted with ease to capture the use of antibiotics in the veterinary, environmental and agricultural sectors. From conception, the developers were keen to ensure that the system should not be costly and should be done once to reduce the human resource constraints peculiar in low resource settings. In terms of the cost, it is estimated to cost about 120,000 Euros to run the system annually, taking into consideration the cost for the soft-ware and cost of payment of salaries of part-time staff. This system will likely be affordable and sustainable if implemented in Africa. The Africa CDC can emulate from the Global PSS example as follows: adapting the same system by recruiting more hospitals in Africa in order to have a more comprehensive picture of antimicrobial consumption in the continent. This should also involve adapting the use of the web based system to capture the veterinary and agricultural sectors in a One Health approach; review the costing to include human resource and logistics specificities in African health systems. It is estimated that for a 500 bed hospital, it would take just 2 weeks to capture all the surveillance data needed. This means even if veterinary and agricultural sectors are included, it would still be a cost effective and sustainable strategy for the AMRSNET to implement; with the availability of online E-modules for training on AMR, there will not be need to spend in organizing costly workshops on AMR. A major challenge for the AMRSNET is how to implement and monitor the progress in a phase based approach. From the deliberations by participants, it was suggested that lessons could be learnt from well-structured approaches such as SLIPTA (Stepwise Laboratory Quality Improvement Towards Accreditation). Based on the ISO 15189:2012(E) standard, the SLIPTA approach uses a checklist that outlines requirements for quality and competency aimed to develop and improve laboratory services towards accreditation [10].

Laboratory mentors who have been trained in the implementation of the checklist are deployed and stationed in laboratories to provide technical support for the laboratories as well as to participate in the training of trainers. This approach has been scaled up in Africa with the support of the US CDC and the Africa Society for Laboratory Medicine (ASLM). A similar system could be adapted for the AMRSNET with the training of AMR mentors who can further train other AMR mentors in clinical, veterinary and agricultural sectors towards the step wise phase based approach. A scorecard that details targets to be met by the different sectors could be adapted for use in monitoring the implementation of this approach. Diagnostics play a crucial role in the control of infectious diseases and in the control of antimicrobial resistance. Researchers, policy makers and implementers really need to be clear on the priority diagnostic needs for antimicrobial resistance surveillance. From our consultations, the following could be considered as diagnostic priorities: the availability of a rapid test to distinguish bacterial and viral infection. The need to ensure the uptake of any of such diagnostics if available in future should be high among clinicians and health personnel.

There are some ongoing efforts. For example, in Tanzania there is the use the e-POCT phone system with biomarkers such as CRP and pro-Calcitonin in reducing inappropriate antibiotic prescription in children. The results of this technology have been encouraging and should be encouraged in other African countries; there is need to select the diagnostic technologies that would be needed for surveillance. For example in Africa, if gram negative bacteria surveillance is chosen, major hospitals could be selected and affordable technologies that detect gram negative bacteria should be considered. If this is implemented, it would be easier to evaluate stewardship programmes aimed at reducing antimicrobial resistance; connectivity with current diagnostics used for AMR. This would help networking and real time implementation of action. Senegal declared a Dengue epidemic on October 24, 2017 and a lot can be learnt and leveraged from their current connectivity capabilities. Using a web based system, notifications about the epidemic can be obtained from cell phones, says Dr Amadou Sall of the Pasteur Institute in Dakar during an informal consultation at the ACDx. The Africa CDC can utilize the same connected system in its AMR network which will be able to be adapted to provide real time information of the strains of bacteria circulating in the network; continuous capacity building in diagnostics to support the AMRSNET. In conclusion, the launch of the Africa CDC AMRSNET is a very important step towards the implementation of its strategic plan on reducing the rate of antimicrobial resistance in Africa. However, lessons must be learnt from ongoing efforts so that we don't re-invent the wheel. Diagnostics certainly play a crucial role in the control of AMR, hence more attention should be given the urgent diagnostic priorities in the continent.

## Competing interests

The authors declare no competing interests.
